# Background Music Dependent Reduction of Aversive Perception and Its Relation to P3 Amplitude Reduction and Increased Heart Rate

**DOI:** 10.3389/fnhum.2019.00184

**Published:** 2019-06-27

**Authors:** Masahiro Matsuo, Fumi Masuda, Yukiyoshi Sumi, Masahiro Takahashi, Atsushi Yoshimura, Naoto Yamada, Hiroshi Kadotani

**Affiliations:** ^1^Department of Psychiatry, Shiga University of Medical Science, Otsu, Japan; ^2^Department of Neuropsychiatry, Keio University School of Medicine, Tokyo, Japan; ^3^Department of Sleep and Behavioral Science, Shiga University of Medical Science, Otsu, Japan

**Keywords:** background music, affective response, mood changes, event-related potentials, modulation of affective perception

## Abstract

Music is commonly used to modify mood and has attracted attention as a potential therapeutic intervention. Despite the well-recognized effects of music on mood, changes in affective perception due to music remain majorly unknown. Here, we examined if the perception of aversive stimuli could be altered by mood-changing background music. Using subjective scoring data from 17 healthy volunteers, we assessed the effect of relaxing background music (RelaxBGM), busy background music (BusyBGM), or no background music (NoBGM) conditions on response to aversive white noise stimulation. Interestingly, affective response to the white noise was selectively alleviated, and white noise-related P3 component amplitude was reduced in BusyBGM. However, affective responses as well as P3 amplitude to reference pure tone stimuli were similar regardless of background music conditions. Interestingly, heart rate (HR) increased in BusyBGM, whereas no increase in HR was found in similar distress, NoBGM condition. These findings suggest that increase in HR, which happens during BusyBGM exposure, can be a reflecting feature of music that ameliorates the affective response to aversive stimuli, possibly through selective reduction in neurophysiological responses.

## Introduction

From majestic operas to a casual humming, music plays an indispensable and extensive role in human life. One reason for the ubiquity of music is its ability to change mood (Sloboda and Juslin, 2001). For example, the choice of background music in a movie can dramatically change the impact of visual scenery perception, even if the music itself is not being consciously listened to Boltz (2004). The mood adjusting effects of background music are not only applicable to movies, but are widely used in environments, such as shopping malls and restaurants, to enhance the behavior of customers (Milliman, 1982, 1986). Background music is not limited to composed melodies: several online music streaming services provide artificial mixtures of daily noise claimed to serve as “concentration helping” background music (Zhang et al., 2013).

Recently, an increasing number of studies have elucidated the efficacy of music therapy (Bradt et al., 2013; Aalbers et al., 2017; van der Steen et al., 2018). Interestingly, a relaxing effect is not the only expected outcome of some music therapies. For example, in music therapy for pain, the main outcome is the alleviation of pain perception (Lee, 2016). This suggests that music can regulate cognitive perception, beyond a direct effect on mood. Another interesting aspect of music therapy is continuous attention to music is not necessary because passive listening is as effective as active listening in several cases (Mercadie et al., 2015; Millett and Gooding, 2018). Indeed, the music used in music therapy is played at normative loudness; it need not be loud or boisterous as in a concert hall. These findings suggest that unconscious listening to background music in daily life can affect mood and consequently modify affective perception.

Such change in affective perception can be assumed as a kind of affective bias. In relation to the clinical consideration, the affective perceptions are negatively biased in patients with depression, which is paralleled by reduced P3 amplitudes related to happy-face perception (Cavanagh and Geisler, 2006). In the basic cognitive science study, it is reported that auditory-induced pleasant mood enhances the cognitive inhibition that is paralleled with pronounced amplitudes in event-related potential (ERP) components between 150 and 550 ms (Yuan et al., 2011). In addition, these early components of ERP are thought to be involved in the mechanism of affective bias (Huang and Luo, 2006). For example, P3 component is related to the valence perception (Conroy and Polich, 2007). Early posterior negativity (EPN) is also considered to be the early stage of affective process, mirroring the fast and effortless detection of emotional stimuli (Olofsson et al., 2008; Ullrich et al., 2016). Consistently, in a recent article, we reported that the sounds of different aversiveness were associated with different neuroelectric activities in this time range. Briefly, aversive white noise stimuli involved more activity in the parietal region than pure tone stimuli in the time range corresponding to EPN and P3 components (Masuda et al., 2018).

Intriguingly, the effects of background music on cognitive function are not conclusive. For example, one study reported that background music has beneficial effect on reasoning or memory performance (Rauscher et al., 1993) whereas another study found detrimental effects on memory and comprehension tasks (Furnham and Strbac, 2002). These contrasting results could be attributable to the difference in the methodologies, targeted cognitive function, or applied choice of music. Choice of music type is important because it is reported that music interferes with the learning process depending on the congruency of the learning material and the kind of background music (Sousou, 1997). Similarly, music with increased arousal and positive affect can improve the performance of certain tests of spatial abilities (Thompson et al., 2001). The difference in the autonomic nervous system (ANS) might be involved in the inconsistent results because the autonomic nervous activity is related to type of music (Zatorre, 2015).

Thus, to establish the basis of affective bias caused by daily loudness music therapy, we performed a multimodal study that examined neurocognitive responses as well as the ANS changes. As an ANS measure that is closely related to mood, we examined changes in heart rate (HR) along with the subjective measurement of mood (Sammler et al., 2007).

We hypothesized that calming music can alleviate the aversive perception paralleled with reduced amplitudes of aversive-related EPN/P3 and reduction of corresponding neural activity in parietal region. In addition, we expected such music to have a soothing effect on ANS activity, such that it would be observable as decreased HR. This report is an extension of our recent work that reported the appraisal mechanism of white noise and pure tone (Masuda et al., 2018).

## Materials and Methods

### Subjects

Advertisements were used to recruit 17 healthy adult participants for this study (10 men; mean age ± standard deviation, 21.6 ± 2.06 years). The participants were compensated with a gift card with a value equivalent to ¥2,500. Interview by a psychiatrist confirmed that the participants had no psychiatric disorders, hearing problems, or smoking history and did not habitually take medication or consume caffeine on the day of the study. All the subjects were right-handed, which was confirmed using Edinburgh handedness score being not <50 (Oldfield, 1971). All subjects had normal hearing ability. Four subjects had experience of taking music lessons in their childhood, but no subject was taking music education at the time of the experiment. This study was in accordance with the recommendations of the ethical committee at Shiga University of Medical Science with written informed consent from all subjects. All subjects gave written informed consent in accordance with the Declaration of Helsinki. The protocol was approved by the ethical committee at Shiga University of Medical Science (Approved #26-227).

### Experimental Design and Settings

#### Background Music and Sound Stimuli

Two types of background music were used to induce changes in mood. Relaxing background music (RelaxBGM) was a privately composed music that was spacious and ethereal, such as music typically played for yoga or meditation. Busy background music (BusyBGM) was an artificial mixture of traffic noise that was reminiscent of a busy highway. The background music was played at the same loudness in all conditions, at an average loudness of 40 dB[A]. In addition, no background music (NoBGM) condition [<30 dB(A) silence] was used.

A 500-ms burst of 50 dB[A] white noise with instantaneous (10 ms) rise/fall times was used as an aversive stimulus. The white noise included all frequency bands within the audible range. A 1,000-Hz pure tone was used as a reference. Pure tone was used as a reference because sounds with 1,000 Hz peaks are most ubiquitously observed (Kim et al., 2012) and less affected by age-related losses in hearing sensitivity (Cruickshanks et al., 1998). We presented the stimuli in a passive task context, where subjects were instructed to simply view a presented fixation point without special attention to sound or background music.

#### Subjective Measures of Mood and Affective Response

The subjects were asked to score their subjective emotional responses using the Self-Assessment Manikin (SAM), a two-dimensional subjective scoring system developed for assessing affective stimuli of the International Affective Picture System (Bradley and Lang, 1994). This is a nine-point rating scale comprising sets of figures to measure valence (1 = unpleasant; 9 = pleasant) and arousal responses (1 = arousing; 9 = calming). We used the Profile of Mood States (POMS), which has high levels of reliability and validity, to measure psychological distress. In the scale, participants were required to rate 65 mood-related adjectives on a 5-point scale (0 = not at all to 4 = extremely). The scores of 65 adjectives were combined to make six sub-scale scores, and Total Mood Disturbance (TMD) was then calculated based on the sub-scale scores. A larger TMD score indicates an increased state of distress.

#### Procedures and Settings

**Figure 1:**
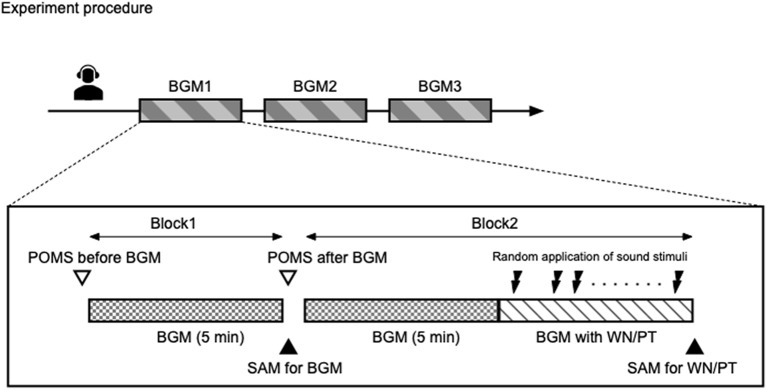
Schematic representation of the study. The study was conducted following randomized counterbalanced crossover design. Each participant underwent three experiments with different background music conditions. In each background music condition, two blocks of experiments were conducted. In block 1, participants were exposed to selected background music for 5 min. In block 2, participants were exposed to the background music for 5 min, followed by exposure to the same background music with intermittent sound stimuli.

Subjects underwent three background music experiments, in which one of the three background music options (RelaxBGM, BusyBGM, or NoBGM) was played (Figure 1, upper panel). The background music was selected following randomized counterbalanced crossover design. During the experiment, subjects remained seated on a chair placed 70 cm in front of a cathode ray tube (CRT) display in a sound proof and electromagnetic shield room. The illumination in the room was maintained at 80 lux. The subjects were instructed to look at a white-cross fixation point that appeared against the black CRT background during the entire experiments. Auditory stimulation was provided through headphones (AKG closed-back headphones, K404, Vienna, Austria).

One experiment was consisted with two major blocks (Figure 1, lower panel). In the first block, participants were only exposed to the background music. The effect of background music on mood was examined by TMD before and after a 5-min exposure to the background music. In addition, the participants were asked to use SAM to rate their appraisal evaluation of the background music. After completion of the ratings, the subjects immediately proceeded to the second block.

In the second block, the participants were exposed to the same background music for 5 min as in the first block, followed by the administration of pure tone and white noise sound stimuli with background music lasting approximately 5 min. The pure tone and white noise sound stimuli were programmed to randomly produce each frequency 75 times, with randomized stimulus intervals of 2,000 ± 200 ms using E-Prime v 2.0 software (Psychology Software Tools, Pittsburgh PA, USA). The participants completed SAM for both white noise and pure tone stimuli immediately after each experiment.

### Electroencephalography Data Acquisition

Electroencephalography (EEG) signals were recorded using NetStation software [Electrical Geodesics Inc (EGI), Eugene, OR, USA] with 64-channel recordings made through a HydrocCel Geodesic Sensor Net v.1.0. gel cap. Data were sampled using a high-input impedance amplifier (200 MΩ, EGI Inc., Model: GES 300), at 500 Hz and referenced to Cz. Electrode impedances were kept at <60 kΩ throughout the experiments, following the guideline recommending the electrode impedance to be less than the input impedance of the amplifier by a factor of at least 100 (Picton et al., 2000). The participants were asked to remain awake, and a vigilant state was qualitatively confirmed by online observation of the EEG signal by a somnologist during the study.

### Event-Related Potential (ERP) Data Processing

EEG data processing was performed using EEGLAB (version 14.1.2; Delorme and Makeig, 2004), an open source toolbox that runs on MATLAB version 2017a (Mathworks Inc. Natick, MA, USA). Briefly, EEG data were re-referenced to the average of the left and right mastoids, and bandpass filtered offline by 0.1–50 Hz using linear finite impulse response filtering method. Gross artifacts were visually rejected following independent component analysis based artifact correction embedded in EEGLAB, excluding 1–3 components produced by eye movement or muscle activity. We epoched all data segments 500 ms prior to and 1,500 ms post stimulations, and baseline corrections were done by subtracting the average of 100 ms prior to stimulation using ERPLAB (version 7.0.0; Lopez-Calderon and Luck, 2014). Epochs for ERP calculation were first selected using the simple voltage threshold function of ERPLAB using 100 μV as the threshold. Finally, an examiner, without the knowledge of the experiment conditions, visually confirmed artifact-free epochs for ERP calculation. The average number of epochs used for ERP calculation was as follows: 55.76 ± 2.57 for pure tone and 53.94 ± 2.44 for white noise in NoBGM; 54.29 ± 2.69 for pure tone and 51.88 ± 2.77 for white noise in RelaxBGM; and 48.59 ± 3.54 for pure tone and 48.76 ± 3.43 for white noise in BusyBGM. To compare component amplitudes, we calculated the mean relative-to-baseline amplitude value between the specified time range from each electrode. To compare ERP component amplitudes on region-of-interest basis, we averaged the potentials of four electrodes (Pz, Cz, C3, and C4). We focused on these four electrodes because EPN/P3 related potentials were most pronounced in these electrodes (Supplementary Figure S1). To investigate the regions involved in the differential processes between pure tone and white noise, we used time series standardized low resolution brain electromagnetic tomography analysis (sLORETA) every 2 ms to estimate the current source density distribution for each ERP component (Pascual-Marqui et al., 1994).

### Heart Rate and Calculation of Autonomic Features

Kubios HRV (version 2.1, Kubios, Finland) was used for HR detection (Tarvainen et al., 2014). The type II lead of electrocardiogram was simultaneously recorded during EEG recordings, and the data were later processed by Kubios HRV. HR was calculated from 20 s data at the beginning or end of BGM exposure.

### Statistical Analyses

Data are shown as mean ± standard error of mean, unless otherwise stated. For the examination of effects by background music and sound stimuli, a two-way repeated measures (3 × 2) analysis of variance (ANOVA) with three background music conditions and two sound stimuli as the within-subjects factors was conducted unless otherwise described. Greenhouse-Geisser correction was used when the sphericity was violated. Bonferroni pairwise comparison was used to adjust for multiple comparisons. SPSS statistics software Version 22 (IBM, Armonk, NY, USA) was used to perform statistical analysis. sLORETA images were statistically compared between sound conditions using the voxel-by-voxel *t*-test, which was corrected by Statistical non-Parametric Mapping (SnPM) randomization (number of randomizations = 5,000). The threshold of statistical significance was set at *P* < 0.05.

### Comparisons of Components of Event-Related Potentials

To examine the changes in sound stimulation related potentials in each background music condition, we focused on 200–300 and 300–450 ms because these time ranges were pivotal in the aversive process of pure tone and white noise (Masuda et al., 2018).

## Results

### Subjective Ratings of Appraisal Response and Consequential Mood by Background Music

**Figure 2:**
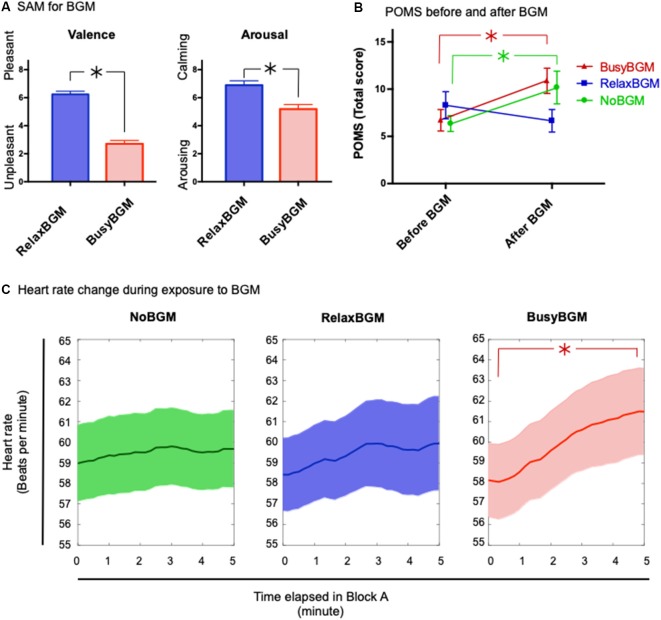
Affective responses and consequential mood for each background music condition. **(A)** Affective responses to relaxing background music (RelaxBGM) and busy background music (BusyBGM) are shown based on two-dimensional evaluation: valence and arousal. Note that RelaxBGM was more pleasant and calming than BusyBGM. **(B)** Emotional consequences of listening to each background music for 5 min are shown. Note that mood worsened in BusyBGM and no background music (NoBGM) conditions, whereas RelaxBGM prevented the worsening. **(C)** Heart rate (HR) changes were noted during exposure to each background music. HR increased in BusyBGM, while HRs at the beginning were similar in all conditions. Asterisk shows significant difference between the indicated pair.

First, to investigate how participants experienced the background music, the two-way repeated measures (2 × 2) ANOVA within the subject factors background music (RelaxBGM and BusyBGM) and subjective evaluations (SAM scores in valence and arousal) was performed. This analysis showed significant effect of background music (*F*_(1,16)_ = 94.298, *P* < 0.01, partial *η*^2^ = 0.855), and SAM (*F*_(1,16)_ = 28.810, *P* < 0.01, partial *η*^2^ = 0.643) as well as their interaction between background music and SAM (*F*_(1,16)_ = 24.800, *P* < 0.01, partial *η*^2^ = 0.608). BusyBGM was perceived as more aversive than RelaxBGM (valence score: BusyBGM, 2.71 ± 0.24; RelaxBGM, 6.24 ± 0.22, Figure 2A, *t*_(16)_ = 13.631, *P* < 0.01). In addition, BusyBGM was more arousing than RelaxBGM (arousing score: BusyBGM, 5.18 ± 0.33; RelaxBGM, 6.88 ± 0.32, *t*_(16)_ = 6.5884, *P* < 0.01).

Thereafter, we examined the mood changes caused by 5-min exposure to RelaxBGM, BusyBGM, and NoBGM using TMD calculated from POMS questionnaire. TMD score had high internal consistency in our study sample (Cronbach’s Alpha = 0.915). The two-way repeated measures ANOVA with background music and the time (before or after background music exposure) as within subject factors found simple main effect by time (*F*_(1,16)_ = 11.437, *P* < 0.01, partial *η*^2^ = 0.417) and no effect of background music (*F*_(2,32)_ = 1.251, *P* = 0.300, partial *η*^2^ = 0.073), and significant interaction between background music and time (*F*_(2,32)_ = 10.159, *P* < 0.01, partial *η*^2^ = 0.388). Following planned comparisons, to check background music specific change of the mood, found worsening of TMD in BusyBGM (pre vs. post: 6.71 ± 1.14 vs. 10.88 ± 1.34, *t*_(32)_ = 4.077, *P* < 0.01, Figure 2B) and NoBGM (pre vs. post: 6.35 ± 0.84 vs. 10.18 ± 1.73, *t*_(32)_ = 3.733, *P* < 0.01), although no worsening was observed in RelaxBGM (pre vs. post: 8.29 ± 1.44 vs. 6.65 ± 1.19, *t*_(32)_ = 1.608, *P* = 0.353).

### Heart Rate Changes Due to the Background Music

Similar to the analysis of the mood changes due to background music, we performed the two-way repeated measures ANOVA on HR with background music and time as within subject factors. We used two representative average HR from two time windows, the first and the last 20 s of BGM exposure (Figure 2C). This analysis found significant main effect of the time (*F*_(1,16)_ = 9.061, *P* = 0.008, partial *η*^2^ = 0.362). However, no significant effect of background music was found (*F*_(2,32)_ = 0.319, *P* = 0.729, partial *η*^2^ = 0.020) and marginal interaction was found (*F*_(2,32)_ = 3.014, *P* = 0.063, partial *η*^2^ = 0.159). In following planned comparisons, an increase in HR was found to be specific to BusyBGM condition (pre vs. post: 58.16 ± 1.79 vs. 61.49 ± 2.10, *t*_(32)_ = 4.291, *P* < 0.01), and HR remained constant in NoBGM (pre vs. post: 58.97 ± 1.86 vs. 59.68 ± 1.89, *t*_(32)_ = 0.901, *P* > 0.900) and RelaxBGM conditions (pre vs. post: 58.43 ± 1.77 vs. 59.94 ± 2.29, *t*_(32)_ = 1.945, *P* = 0.182).

### Changes in Affective Response to White Noise/Pure Tone in the Three Background Music Conditions

**Figure 3:**
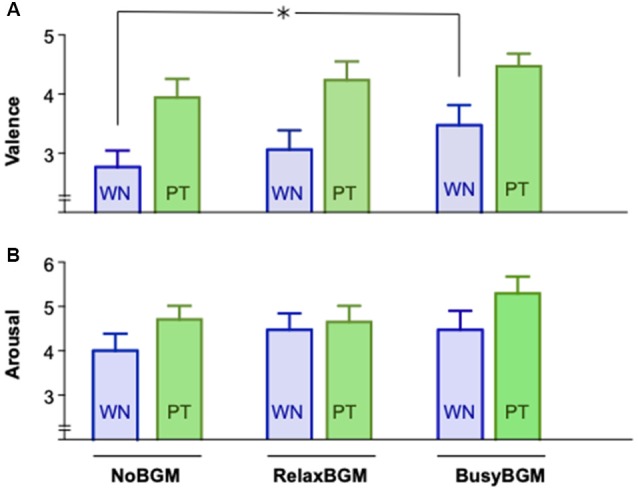
Affective response to white noise and pure tone for each background music condition. Self-Assessment Manikin (SAM) scores for white noise and pure tone are graphically presented. Panel **(A)** shows the valence scores, whereas Panel **(B)** shows the arousal scores. Significant difference due to background music condition is shown by asterisk.

We examined if pure tone and white noise caused different affective responses in the three background music conditions using the two-way repeated measures ANOVA. The analysis on valence found significant main effect for the sound stimuli (*F*_(1,16)_ = 16.812, *P* = 0.001, partial *η*^2^ = 0.512) and marginal significance for background music (*F*_(2,32)_ = 2.744, *P* = 0.079, partial *η*^2^ = 0.146), although the interaction between them was not significant (*F*_(2,32)_ = 0.232, *P* = 0.794, partial *η*^2^ = 0.014). Planned comparisons within the same sound stimulus found aversive response to white noise significantly reduced in BusyBGM compared to those in NoBGM (BusyBGM vs. NoBGM: 3.47 ± 0.34 vs. 2.76 ± 0.28, *t*_(32)_ = 3.339, *P* < 0.01, Figure 3A), although no significant reduction of aversiveness was found in RelaxBGM compared to NoBGM (RelaxBGM vs. NoBGM: 3.06 ± 0.33 vs. 2.76 ± 0.28, *t*_(32)_ = 1.391, *P* = 0.521). Interestingly, aversive response to pure tone was comparable to NoBGM in both RelaxBGM (RelaxBGM vs. NoBGM: 4.24 ± 0.32 vs. 3.94 ± 0.31, *t*_(32)_ = 1.391, *P* = 0.521) and BusyBGM (BusyBGM vs. NoBGM: 4.47 ± 0.21 vs. 3.94 ± 0.31, *t*_(32)_ = 2.504, *P* = 0.052).

The same analysis on arousal found marginal effect of sound stimuli (*F*_(1.000,16.000)_ = 4.176, *P* = 0.058, partial *η*^2^ = 0.207) but no significant effect of background music (*F*_(1.916,30.656)_ = 1.171, *P* = 0.322, partial *η*^2^ = 0.068) as well as no significant interaction (*F*_(1.471,23.539)_ = 2.368, *P* = 0.127, partial *η*^2^ = 0.129).

Planned comparisons found no significant difference in arousal responses to white noise, which was similar to NoBGM condition in both RelaxBGM (RelaxBGM vs. NoBGM: 4.47 ± 0.37 vs. 4.00 ± 0.38, *t*_(16)_ = 1.095, *P* = 0.869) and BusyBGM (BusyBGM vs. NoBGM: 4.47 ± 0.43 vs. 4.00 ± 0.38, *t*_(16)_ = 1.000, *P* > 0.900 in BusyBGM, Figure 3B). Similarly, comparable arousal responses were found for pure tone (arousal response to pure tone: RelaxBGM vs. NoBGM: 4.65 ± 0.36 vs. 4.71 ± 0.31, *t*_(16)_ = 0.203, *P* > 0.900) and in BusyBGM (BusyBGM vs. NoBGM: 5.29 ± 0.38 vs. 4.71 ± 0.31, *t*_(16)_ = 2.163, *P* = 0.138).

### Neurophysiological Response to Sound Stimulations

**Figure 4:**
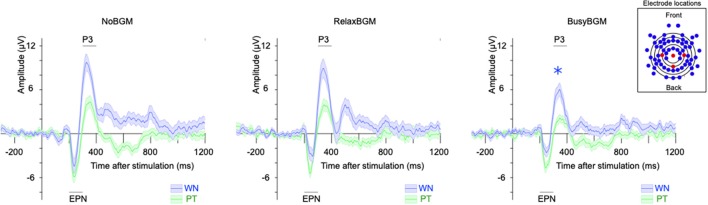
Event-related potentials (ERPs) to white noise and pure tone for each background music condition. Each panel shows the grand average ERP for each BGM condition. ERPs evoked by white noise (blue line), and pure tone (green line) are presented. Shaded colors represent the mean ± standard error. (The inset box) The electrodes used in the calculation of ERP (Pz, Cz, C3, and C4) are shown in red, while others are in blue.

The two-way ANOVA analysis on P3 component amplitude found simple main effect of background music (*F*_(2,32)_ = 7.601, *P* < 0.01, partial *η*^2^ = 0.322) and sound stimuli (*F*_(1,16)_ = 77.962, *P* < 0.01, partial *η*^2^ = 0.830), although there was no significant interaction (*F*_(2,32)_ = 0.929, *P* = 0.405, partial *η*^2^ = 0.055). Following comparison within each sound stimuli found that white noise-related amplitude was significantly smaller in BusyBGM (BusyBGM vs. NoBGM: 3.36 ± 0.58 vs. 5.64 ± 0.64 μV, *t*_(32)_ = 3.906, *P* < 0.01; Figure 4), although amplitude was comparable between RelaxBGM and NoBGM (RelaxBGM vs. NoBGM: 4.54 ± 0.42 vs. 5.64 ± 0.64 μV, *t*_(32)_ = 1.885, *P* = 0.21). Interestingly, the pure tone–related amplitude in BusyBGM was comparable to NoBGM (BusyBGM vs. NoBGM: 0.98 ± 0.34 vs. 2.15 ± 0.58 μV, *t*_(32)_ = 1.999, *P* = 0.16) as well as in RelaxBGM (RelaxBGM vs. NoBGM: 1.47 ± 0.42 vs. 2.15 ± 0.58 μV, *t*_(32)_ = 1.175, *P* = 0.75).

For EPN component, simple main effect of sound stimuli (*F*_(1,16)_ = 31.617, *P* < 0.01, partial *η*^2^ = 0.664) was found, although there was no main effect of background music (*F*_(2,32)_ = 0.361, *P* = 0.700, partial *η*^2^ = 0.022) and no significant interaction (*F*_(2,32)_ = 0.591, *P* = 0.560, partial *η*^2^ = 0.036). Planned comparisons of amplitudes within each sound stimuli found EPN in BusyBGM (BusyBGM vs. NoBGM: −0.64 ± 0.38 vs. −0.89 ± 0.72 μV, *t*_(32)_ = 0.587) and RelaxBGM (RelaxBGM vs. NoBGM: −1.17 ± 0.69 vs. −0.89 ± 0.72 μV, *t*_(32)_ = 0.634) were comparable to those in NoBGM for white noise. Pure tone–related EPN in BusyBGM (BusyBGM vs. NoBGM: −2.69 ± 0.40 vs. −3.35 ± 0.60 μV, *t*_(32)_ = 1.524, *P* = 0.412) and RelaxBGM (RelaxBGM vs. NoBGM: −2.96 ± 0.45 vs. −3.35 ± 0.60 μV, *t*_(32)_ = 0.890, *P* > 0.900) were also comparable to those in NoBGM.

### Source Localization of ERP

**Figure 5:**
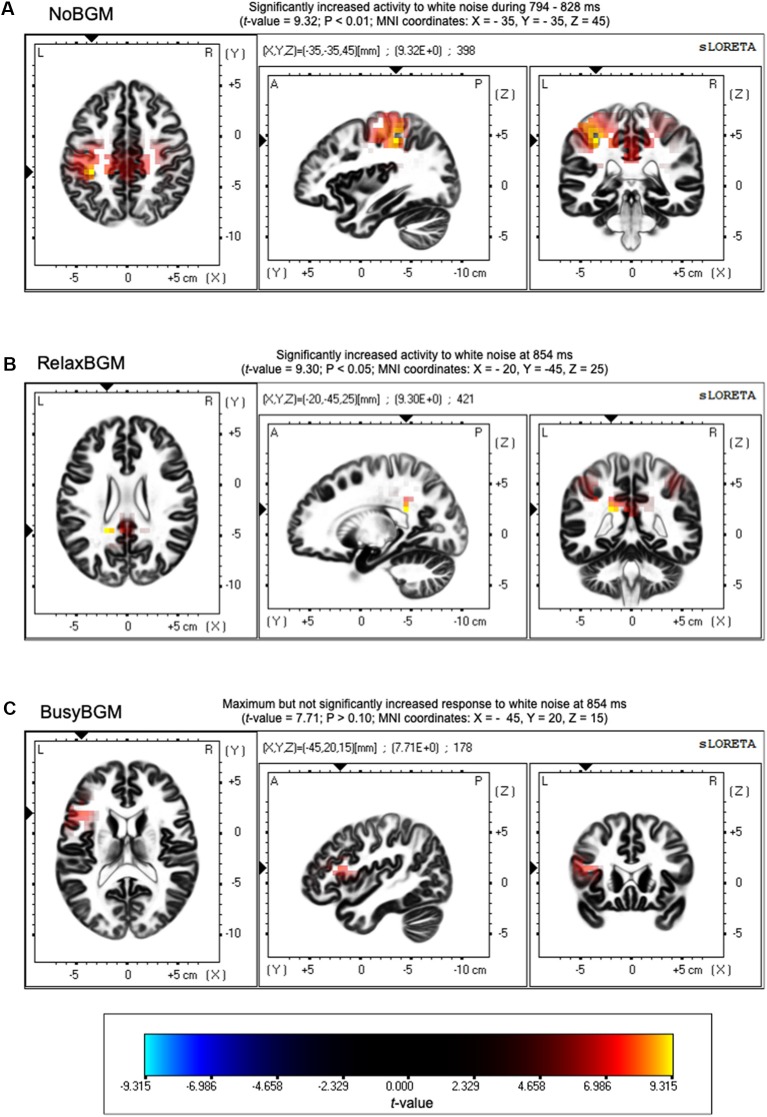
Source localization of differences in ERPs. Differences in neuroelectric activity induced by white noise and pure tone are presented. Regions with significantly increased neuroelectric activity responding to white noise stimulus are shown in yellow to red gradations. **(A)** Increased activities were found around Brodmann area 40 in no background music (NoBGM) condition. **(B)** Increased activities were found around Brodmann area 31 in relaxing background music (RelaxBGM) condition. **(C)** No statistically different responses were found in busy background music (BusyBGM) condition. Color bars show the locations of extreme *t*-values that close to the significant level (*P* < 0.05, corrected for multiple comparisons). Maximum *t*-value and corresponding *P*-value are described above in corresponding figures, and the regions of maximum difference are shown as Montreal Neurological Institute (MNI) coordinates.

Time series analysis using sLORETA between white noise and pure tone revealed significantly greater electrical activity induced by white noise than pure tone under NoBGM and RelaxBGM, whereas no difference was found for BusyBGM (Figure 5). In the NoBGM condition, significant difference between white noise and pure tone were found for the time window between 294 and 328 ms after sound stimulation, as previously reported (Masuda et al., 2018). During this time range, significantly increased electrical activity was found in the parietal lobe centering at the left inferior parietal lobule, for white noise compared with pure tone (left parietal lobe, BA 40, Figure 5A). In RelaxBGM, the difference between white noise and pure tone at the same time range was not found. However, there was a significantly increased electrical activity in the posterior cingulate cortex (PCC) in white noise compared with pure tone (Brodmann 40) at 340 ms in RelaxBGM (Figure 5B).

## Discussion

In this study, we examined the effect of mood-changing background music on affective perception. Because music therapy is reportedly used in pain clinics to reduce pain perception, we predicted that calming background music would help to reduce aversive reaction to white noise.

We used two newly composed background music, aiming to change the mood. The initial assessment of appraisal response to RelaxBGM and BusyBGM revealed that they had intended appraisal effects. As we expected, BusyBGM had mood worsening effect. Slightly different from our initial expectation, the mood was worsened in NoBGM condition possibly because of the stress of sitting still. RelaxBGM appeared to prevent such mood worsening, if not improved the mood. The mood in BusyBGM or NoBGM was not at an evidently stressful level, as the POMS total disturbance scores in the present study were low compared to that in studies assessing a stressful condition using the same measure (Rosenzweig et al., 2003). Intriguingly, even with the similar level of mood worsening in BusyBGM and NoBGM, ANS activity selectively changed in BusyBGM as shown by the increased HR. Considering that the initial HR was similar at the beginning of background music exposure, HR increase was specifically attributable to BusyBGM. The result is consistent with a previous study that showed HR changes depend on music (Koelsch and Jäncke, 2015), particularly in the presence of discomforting music (Sammler et al., 2007). Thus, the absence of HR increase in the NoBGM condition suggests that ANS activity could be differentially modulated even in the same subjective distress level. This is consistent with a report that showed differential ANS modulation by different type of stressors (Hu et al., 2016).

In examination of the background music effect on affective perception of white noise and pure tone, we found unexpected reduction in aversive response to white noise in BusyBGM. The reduction was specific to white noise, suggesting that low level aversiveness, as that found against pure tone, was less prone to the background music effect. One possible psychological explanation for these unexpected results is that moderate distress can reduce the effect of aversive stimulation. It is often indicated that moderate stress is more facilitating for human performance than no stress because the presence of stress often leads to improved performance (Smeets et al., 2008; Hupbach and Fieman, 2012) and emotion (Marin et al., 2010).

In this study, the neurophysiological responses showed the effect of background music similar to subjective response: white noise-related P3 amplitude was reduced in BusyBGM, whereas that in RelaxBGM remained comparable to NoBGM. The reduction in P3 amplitudes in BusyBGM was not due to simple phonic masking effects of the stimulus sounds by background music because the same loudness RelaxBGM did not show the same effects. In addition, P3 amplitude related to pure tone was comparable in all background music, suggesting that the observed amplitude change was white noise-specific. The reduction in P3 amplitude could be assumed as a reflection of reduced cognitive capacity to the sound stimulation, according to the processing capacity model (Kok, 2001). A study using a similar sound stimulation technique reported reduced ERP amplitudes in a state with increased mental concentration (Ullsperger et al., 2001). The amplitude reduction in sound-related ERP due to mental state is reminiscent of distress-dependent ERP changes in the present study. Thus, it is suggested that passive hearing of BusyBGM continuously consumes cognitive capacity, thereby reducing the white noise-related P3 component, although RelaxBGM did not have such an effect. In addition, a similar sound probe experiment showed the P3 amplitude related to startling loud sound was reduced while subjects were looking at emotional pictures (Keil et al., 2007). Because our study used normative loudness sound (50 dB) stimulation, instead of loud sound (95 dB) as in Keil’s study, the present results expand the knowledge that appraisal response to everyday level loudness sound could also be modulated by background music. Contrary to our findings, a study addressing the effects of similar background music conditions (excited background music, relax background music, and NoBGM) on cognitive inhibitory function reported that N2d and P3 component amplitudes are not affected (Burkhard et al., 2018). These findings suggest that the interference of background music might be a cognitive function specific, although this idea awaits further examination.

The time range corresponding to P3 (300–650 ms) is thought to be involved in linking sound stimuli and emotion (Koelsch, 2010). Our previous findings also support the involvement of P3 in the valence determination for white noise perception (Masuda et al., 2018). To further examine the mechanism of reduced P3 amplitude, we performed the current source density analysis on aversive perception related potentials. This analysis showed significantly different neuroelectric activities between white noise and pure tone in NoBGM and RelaxBGM, whereas no significant difference was found in BusyBGM.

The absence of significantly different neuroelectric activity in BusyBGM was consistent with the decreased P3 amplitude difference in BusyBGM, whereas significantly different neuroelectric activities were consistent with the significant ERP amplitudes difference in NoBGM and RelaxBGM. The increased neuroelectric activity of the parietal region in NoBGM may have resulted from the additional process associated with white noise, presumably resolving the sound feature (Masuda et al., 2018). In RelaxBGM, increased neuroelectric activity was found in the PCC. The PCC is generally believed to function as one of the nodes in default mode network (Buckner et al., 2008), and thus, increased activity in white noise compared with pure tone process is unexpected. However, a report mentioned that the PCC also plays a role in the cognitive perception of tintius (Vanneste and De Ridder, 2012), suggesting that the activity in the area may be involved in the perception of discomfort auditory experience. These results suggested that background music exert effects on process later than 300 ms, explaining why we did not find background music effect on EPN.

Considering that the HR increase was the difference between NoBGM and BusyBGM, HR increase could be a physiological feature predicting reduced perception of aversiveness. Because increased HR is associated with increased mental workload (Liu et al., 2017), HR increase in BusyBGM may reflect increased mental workload related to continuous auditory processing of busy noise. This assumption fits aforementioned cognitive capacity model. Thus, the P3 amplitude reducing effect may be exerted by background music that accompanies an increase in HR possibly through increased mental activity, which was typically found in BusyBGM in this study.

Although we have discussed the results of the present study in relation to music and possible link to music therapy in general, there are several limitations. First, we used white noise as aversive stimulation, although it was only shown to be aversive relative to 1,000 Hz pure tone. Thus, we should be careful when interpreting the findings of the present study as a common mechanism underlying all aversive stimuli. In addition, we used two background music, as a representative of busy music and relaxing music. However, it is not warranted that all music within one category will have the same effect. In fact, RelaxBGM did not improve the mood in this study, although it apparently prevented mood worsening. Thus, it is inappropriate to conclude the effect of the mood improving music based on the results of the present study. It should also be noted that we used city noise as BusyBGM. Further studies in this field are required to generalize the effects of background music on affective perception. Regarding the statistics, we reported the results of planned comparisons in all analyses based on the suggestion that comparisons are meaningful even when interaction was not significant (Wei et al., 2012). Last, our study sample included four participants who had history of music education. We included these participants to keep statistical power, but we also confirmed that results were largely similar even after excluding these participants (Supplementary Material).

Overall, this study concluded that background music can modulate affective perception *via* changes in neuroelectric response to certain stimulation. We hope these findings facilitate the optimization of music therapy and cognitive control.

## Ethics Statement

All subjects gave written informed consent in accordance with the Declaration of Helsinki. The protocol was approved by the ethical committee at Shiga University of Medical Science (Approved #26-227).

## Author Contributions

HK and MM conceived the study. FM and YS recruited participants and conducted the study, under management by NY. AY conducted the mathematical analysis, and MT conducted the statistical analysis. MM prepared the manuscript and HK confirmed the final version of the manuscript.

## Conflict of Interest Statement

Background music source was provided by YAMAHA corporation. The authors declare that the research was conducted in the absence of any commercial or financial relationships that could be construed as a potential conflict of interest.

## Abbreviations

NoBGM: no background music
RelaxBGM: relaxing background music
BusyBGM: busy background music
ANOVA: analysis of variance
ANS: autonomic nervous system
CRT: cathode ray tube
HR: heart rate
POMS: Profile of Mood States
TMD: Total Mood Disturbance
SAM: Self-Assessment Manikin
ERP: event-related potential
EPN: early posterior negativity
LORETA: low resolution brain electromagnetic tomography
PCC: posterior cingulate cortex.
