# Supplementary material for: Background Music Dependent Reduction of Aversive Perception and Its Relation to P3 Amplitude Reduction and Increased Heart Rate
Source: Front Hum Neurosci. 2019 Jun 27;13:184. doi: 10.3389/fnhum.2019.00184 (PMC6610262; doi:10.3389/fnhum.2019.00184)
Supplement: Supplementary file 1 [file Data_Sheet_1.PDF]

Supplementary document 1

To examine the effect of past music education we conducted a repeated-measures ANOVA using data from 13 participants who had no history of music education.

EPN amplitude

We performed the two-way repeated measures ANOVA with background music and sound stimuli as within subjects factors on EPN amplitude. This analysis found no effect of background music [$F(2, 24) = 0.914$, $P = 0.414$, partial $\eta 2 = 0.071$] but significant effect of sound stimuli [$F(1, 12) = 26.804$, $P < 0.01$, partial $\eta 2 = 0.691$]. There was no interaction between background music and sound stimuli [($F(2, 24) = 0.262$, $P = 0.772$, partial $\eta 2 = 0.021$]. Following planned comparisons found no significant difference in white noise related EPN amplitude depending on background music conditions. Similarly no significant background music dependent difference for pure tone related EPN.

P3 amplitudes

The same two-way repeated measures ANOVA on P3 amplitude found significant main effect of both background music [$F(1.387, 16.643) = 5.793$, $P = 0.02$, partial $\eta 2 = 0.326$] and sound stimuli [$F(1.000, 12.000) = 55.193$, $P < 0.01$, partial $\eta 2 = 0.821$], although interaction was not found [($F(1.381, 16.577) = 1.473$, $P = 0.252$, partial $\eta 2 = 0.109$]. Following planned comparisons found white noise related P3 amplitude in BusyBGM was significantly smaller than that in RelaxBGM (0.95 ± 0.30 vs 4.66 ± 0.53 μV for BusyBGM vs RelaxBGM). Also, white noise related P3 amplitude in BusyBGM was almost significantly smaller than that in NoBGM (0.95 ± 0.30 vs 6.01 ± 0.79 μV for BusyBGM vs NoBGM). However, there was no significant background music dependent difference for pure tone related P3.
