# Supplementary figures and images for: Background Music Dependent Reduction of Aversive Perception and Its Relation to P3 Amplitude Reduction and Increased Heart Rate
Source: Front Hum Neurosci. 2019 Jun 27;13:184. doi: 10.3389/fnhum.2019.00184 (PMC6610262; doi:10.3389/fnhum.2019.00184)

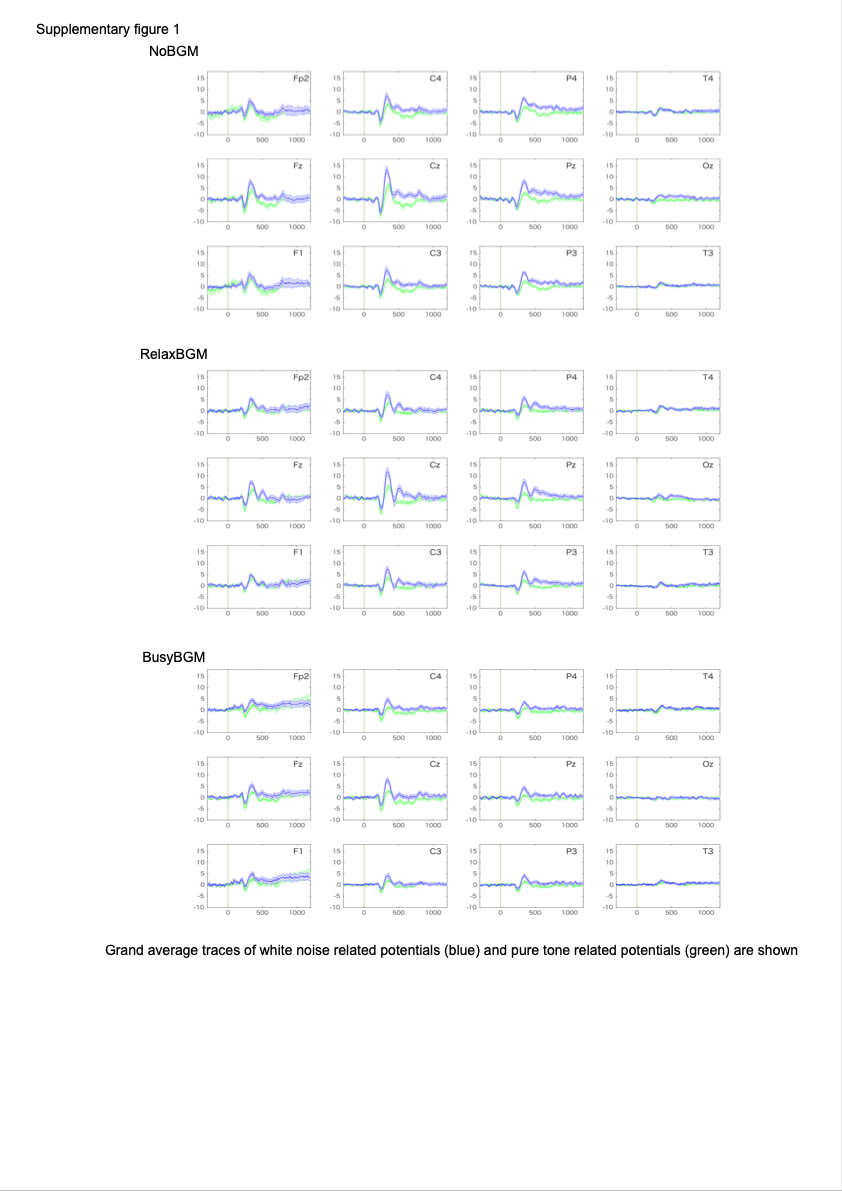

Supplement: Supplementary file 2 [file Image_1.TIFF]
